# Clinical analysis of hepatic angioleiomyoma: Two case reports

**DOI:** 10.1097/MD.0000000000014661

**Published:** 2019-03-15

**Authors:** Bin Jiang, Qiu-Ni Chen, Fu-Zhen Qi, Jian-Bo Xu, Ya-Bin Yu, Yan Song

**Affiliations:** a Department of Hepatobiliary and Pancreatic Surgery; b Department of Hematology, The Affiliated Huai’an No. 1 People's Hospital of Nanjing Medical University, Huai’an, Jiangsu Province, PR China.

**Keywords:** angioleiomyoma, case report, liver hemangioma, vascular leiomyosarcoma

## Abstract

**Rationale:**

Angioleiomyoma is an uncommon benign tumor that originates from the vascular smooth muscle cells and contains thick-walled vessels. It can appear anywhere in the body but more frequently in the extremities (especially in the lower limbs) and rarely invades the internal organs.

**Patient concerns:**

A 52-year-old Chinese woman was referred to our hospital because of finding liver neoplasm 2 weeks ago (case first) and a 64-year-old Chinese woman was admitted to hospital with enlargement of the hepatic neoplasm revealed in follow-up, who was diagnosed with angioleiomyoma of left kidney 2 years ago (case second).

**Diagnosis:**

All patients were diagnosed with hepatic angioleiomyoma by pathological results.

**Interventions:**

All patients received surgical treatment, with laparoscopic hepatectomy of the IVb segment in case 1 and laparoscopic hepatic left lateral lobectomy in case 2.

**Outcomes:**

The 2 patients have eventually recovered, and no recurrences or other complications have been observed so far.

**Lessons:**

Because of atypical clinical symptoms, no specificity in laboratory examination, and lack of characteristic imaging findings, angioleiomyoma is easily misdiagnosed for another disease of the liver. But with complete resection, the prognosis is generally good.

## 1 Introduction

Angioleiomyoma is an infrequent benign tumor and a rare version of leiomyoma, originating from smooth muscle cells and containing thick-walled vessels.^[1–8]^ It may occur anywhere in the body but more frequently in the extremities (especially the lower limbs) and rarely in other parts of the body.^[1,6]^ There are very few literatures reporting the angioleiomyoma of the liver.^[1]^ Angioleiomyoma of extremities appears as dermal or subcutaneous nodule more often .^[1,6,7]^ The clinical manifestations of angioleiomyoma in the internal organs are related to the location and size of the tumor, including abdominal pain, mass, and bleeding. In this study, 2 cases of hepatic angioleiomyoma were presented. And more particularly, 1 patient had angioleiomyoma of the left kidney, 2 years ago.

## 2 Ethical review

Ethical review was nonessential because this case report did not violate patient's privacy. And written informed consent was provided by all patients.

## 3 Case 1

**Figure 1:**
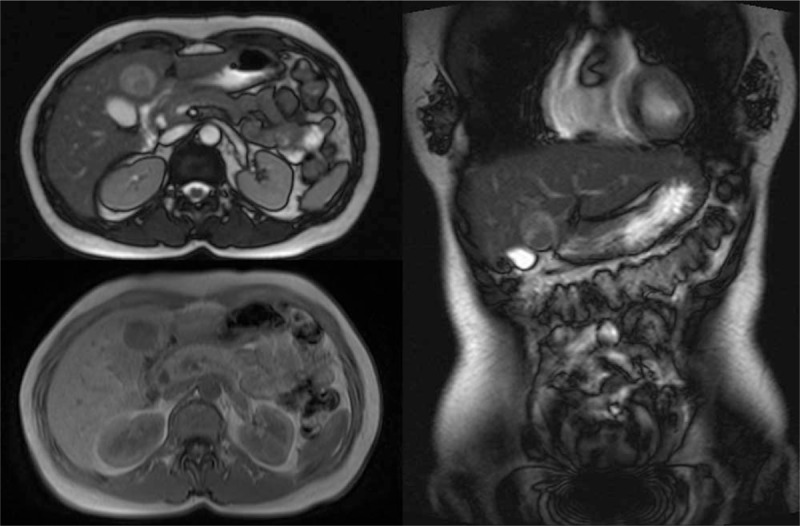
The magnetic resonance imaging (MRI) examination of the upper abdomen showed a well-defined, 30×29 mm^2^ approximately, a nearly-circular shadow in the IVb segment of the liver, with low signal intensity on T1W imaging and slightly high signal intensity on T2W imaging.

**Figure 2:**
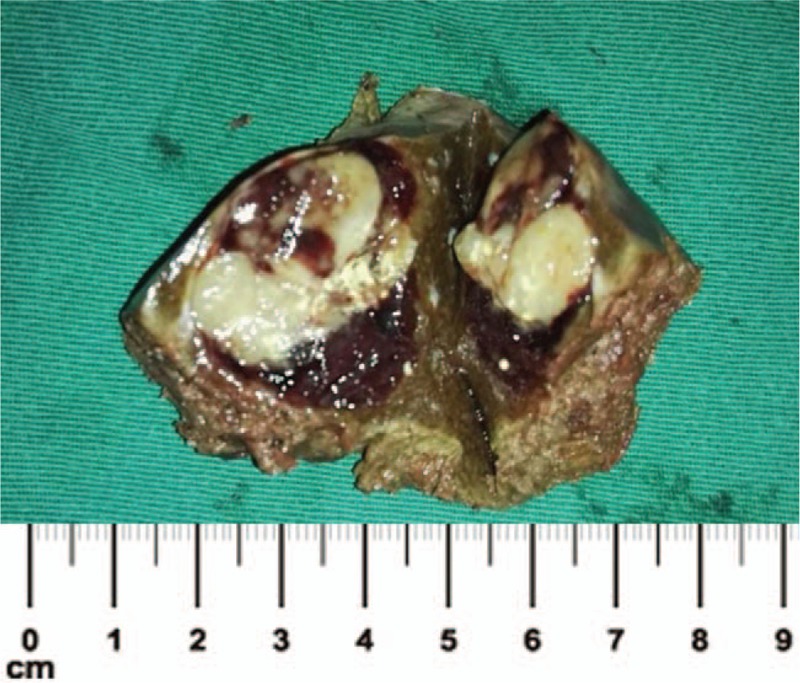
The liver mass after postoperative.

**Figure 3:**
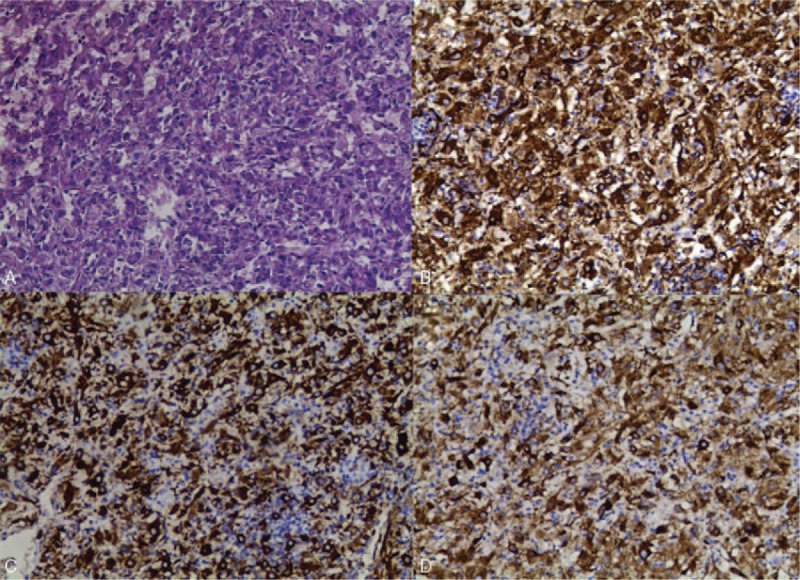
A, Angioleiomyoma tissue stained with H&E (magnification, ×40); angioleiomyoma tissue immunohistochemical stained SMA (B), Melan-A (C), HMB45 (D) (magnification, ×40).

A 52-year-old Chinese woman was hospitalized because of liver mass diagnosed 2 weeks ago. The magnetic resonance imaging (MRI) examination of the upper abdomen showed a well-defined, 30 × 29 mm^2^ approximately, a nearly-circular shadow in the IVb segment of the liver, with low signal intensity on T1W imaging and slightly high signal intensity on T2W imaging (Fig. 1). After the hospitalization, the laboratory findings revealed that human anti-hepatitis B surface antibody (HBsAb), human anti-hepatitis B virus e antibody (HBeAb), and human anti-hepatitis B core antibody (HBcAb) were positive. Because of the MRI results considering liver tumor, the laparoscopic hepatectomy of the IVb segment was implemented. The liver neoplasm removed was about 3.0×3.0×3.0 cm^3^ (Fig. 2). The pathological examination reported that the neoplastic cell was arranged in a nest-like pattern, in which a small number of lipoid cells could be seen. And the results of immunohistochemical analysis showed as follows: Hepatocyte (Negative), Ki- 67 (approximately 1% Positive), CK (Negative), CK 19 (Negative), HMB45 (Positive), Melan-A (Positive), S-100 (Negative), Desmin (Negative), SMA (Positive), Caldesmon (focal Positive), GPC-3 (Negative), GS (focal Positive) (Fig. 3). According to pathological findings a large number of smooth muscle fiber bundles, with abundant thick-walled vascular structures, were observed without nuclear atypia or significant mitosis which was commonly seen in malignant cells, and immunohistochemical staining that HMB45 (Positive), Melan-A (Positive), SMA (Positive), the liver neoplasm was diagnosed ultimately as hepatic angioleiomyoma. Four months after discharge, the patient was reported no recurrence of angioleiomyoma and any postoperative complications.

## 4 Case 2

**Figure 4:**
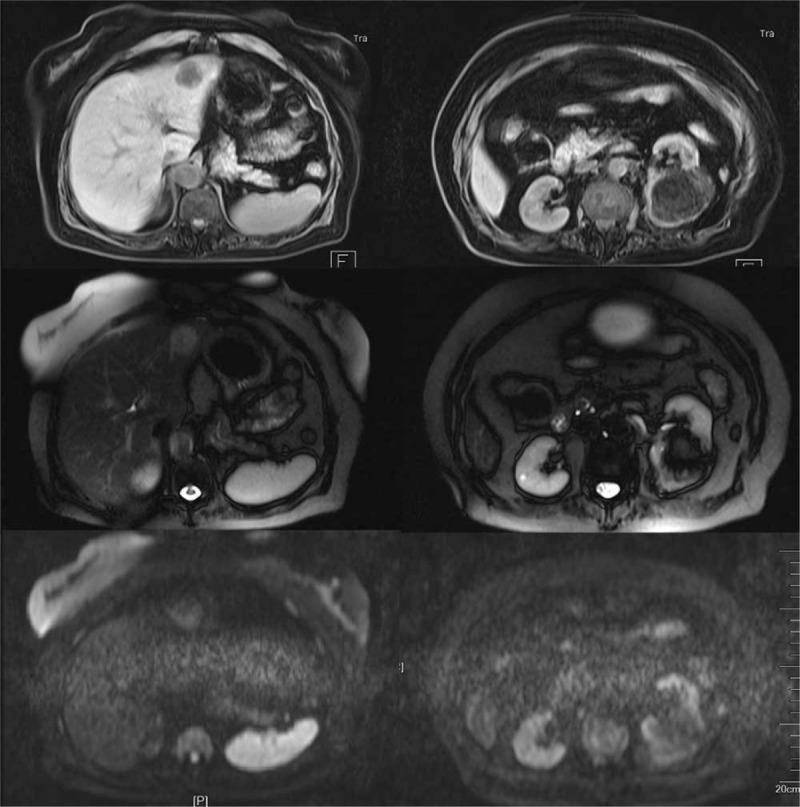
The MRI examination showed an approximately 8.4 × 5.8 cm well-demarcated mass with mixed signals in the lower pole of the left kidney, with low signal intensity on T1W imaging and slightly high signal intensity on T2W imaging. The masses of the left kidney considered to be an angiomyolipoma and the masses of liver need to exclude the possibility of a metastatic tumor. MRI = magnetic resonance imaging.

**Figure 5:**
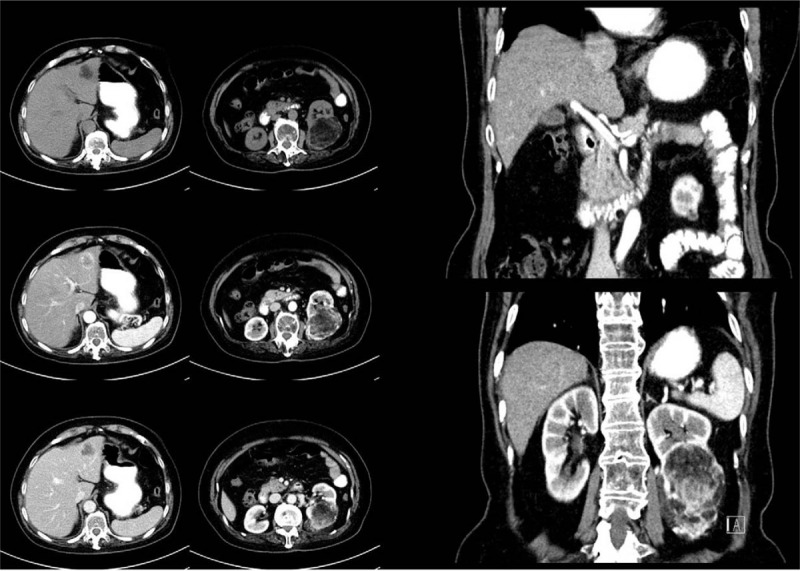
The computed tomography (CT) examination revealed a lobulated low-density soft tissue mass in the left liver, showing a ring-shaped enhancement at the edge of the mass in the enhanced scanning arterial phase and a lower enhanced density than the surrounding liver tissue in the venous phase and the parenchymal phase. And under the left kidney, an irregular mixed density tumor was found, which contained fat density, flocculent soft tissue density, and calcified nodules. The soft tissue was obviously strengthened during the enhanced scanning, and large vessels were visible at the margin and inside of the tumor.

**Figure 6:**
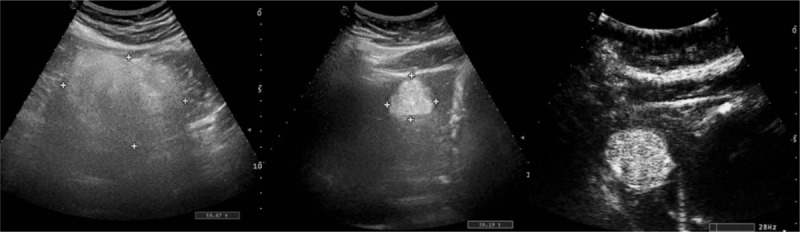
The contrast-enhanced ultrasound examination, which displayed that the blood supply of the left kidney mass is rich and the enhancement is uneven, presenting a “fast-forward and fast-out” mode.

**Figure 7:**
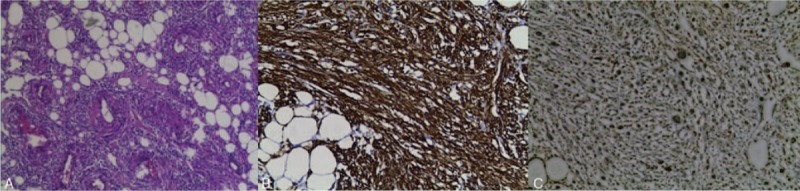
A, Angioleiomyoma tissue stained with H&E (magnification, ×40); angioleiomyoma tissue immunohistochemical stained SMA (B), HMB45 (C) (magnification, ×40).

A 64-year-old Chinese woman was admitted to hospital with symptoms of left lumbago and dorsalgia 2 years ago. First, the B-type ultrasonic inspection showed left kidney lesions. Then the MRI examination showed an approximately 8.4×5.8 cm well-demarcated mass with mixed signals in the lower pole of the left kidney, with low signal intensity on T1W imaging and slightly high signal intensity on T2W imaging (Fig. 4). The mass of the left kidney was considered to be an angiomyolipoma and the mass of liver needed to exclude the possibility of a metastatic tumor. The computed tomography (CT) examination revealed a lobulated low-density soft tissue mass in the left liver, showing a ring-shaped enhancement at the edge of the mass in the enhanced scanning arterial phase and a lower enhanced density than the surrounding liver tissue in the venous phase and the parenchymal phase. And in the lower left kidney, an irregular mixed density tumor was found, which contained fat density, flocculent soft tissue density, and calcified nodules. The soft tissue was obviously strengthened during the enhanced scanning, and large vessels were visible at the margin and inside of the tumor (Fig. 5). Moreover, we also performed contrast-enhanced ultrasonography, showing that the left kidney mass was rich in blood supply and was unevenly enhanced, presenting a “fast-forward and fast-out” mode, which was considered to be kidney cancer. The mass of the liver was rich in blood supply, showing a “fast-forward” mode, which was considered to be metastatic tumor (Fig. 6). Ultimately, after consultation with urology department, hepatobiliary surgery department, medical imaging department, and oncology department, a suggestion was made that the left kidney was radically removed. Then choose surgery or regular follow-up of the liver mass based on pathological results of the kidney's mass. The kidney mass removed was 6.0×0.8×2.0 cm^3^. The pathological examination suggested that many blood vessels were visible in the mass, as a small-diameter fissure surrounded by many differentiated smooth muscle cells and some adipose tissue. And the results of immunohistochemical analysis showed as follows: HMB45 (Positive), CD10 (Negative), RCC (Negative), PAX-2 (Negative), CK (Negative), Ki-67 (< 1% Positive), S-100 (Negative), SMA (Positive), Vim (Positive) (Fig. 7). The pathological examination suggested that abundant thick-walled vascular structures in the mass were mixed with a large number of smooth muscle fiber bundles, and no obvious atypia or significant mitosis of malignant tumor cells was observed. Based on these, the renal mass was diagnosed as angioleiomyoma. The liver mass was not eventually removed, but a regular radiographic examination was selected.

**Figure 8:**
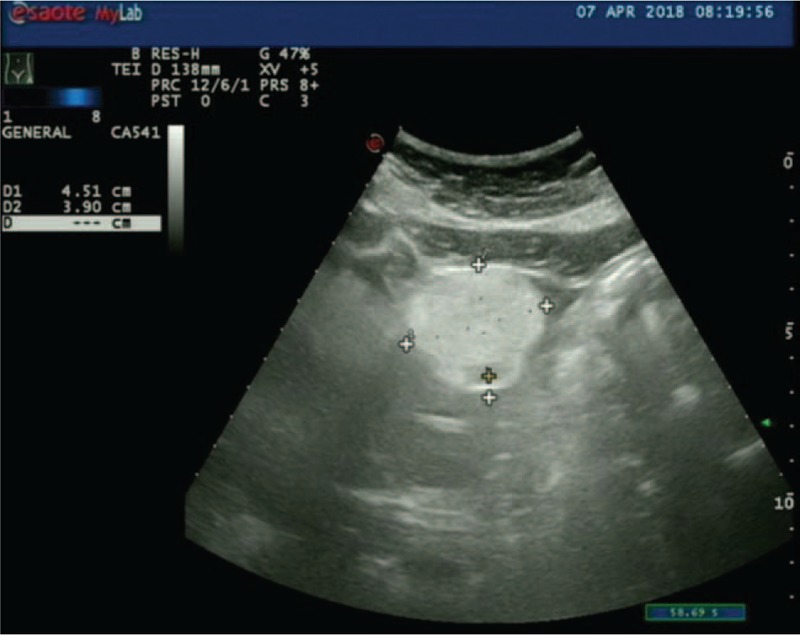
The B-type ultrasonic inspection showed that a hyperechoic nodule can be seen in hepatic left lateral, about 4.5×3.9 cm^2^ in size, with a clear boundary, which is considered a hepatic hemangioma.

**Figure 9:**
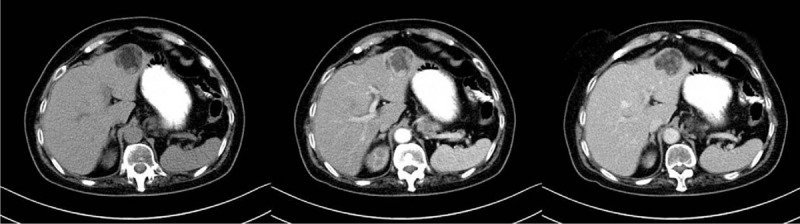
The computed tomography (CT) examination showed that in the left hepatic lobe, a kind of nearly-circular low density with a little fat solid density shadow can be seen, with a maximum diameter of 3.8 cm. After enhancement, the mass appeared to be significantly unevenly enhanced in the arterial phase and reduced in the venous phase and the delayed phase, which was considered primary hepatic carcinoma.

**Figure 10:**
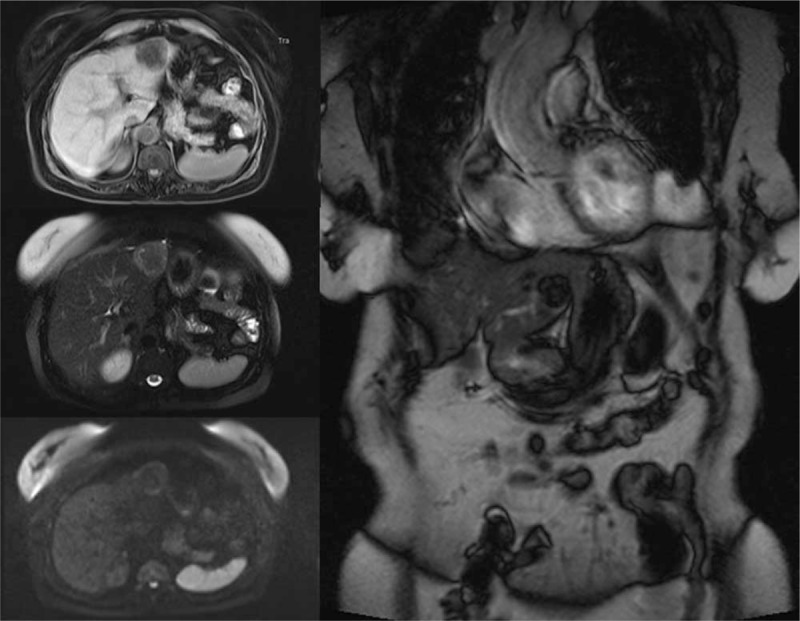
The MRI reported that a nearly-circular abnormal signal shadow, about 4.4×4.2 cm^2^ in size, was seen in hepatic left lateral, presenting a lamellar high signal in phase, a low signal out phase, and an equal or low signal in T2 fat-suppressed imaging, which is considered liver cancer with steatosis. MRI = magnetic resonance imaging.

**Figure 11:**
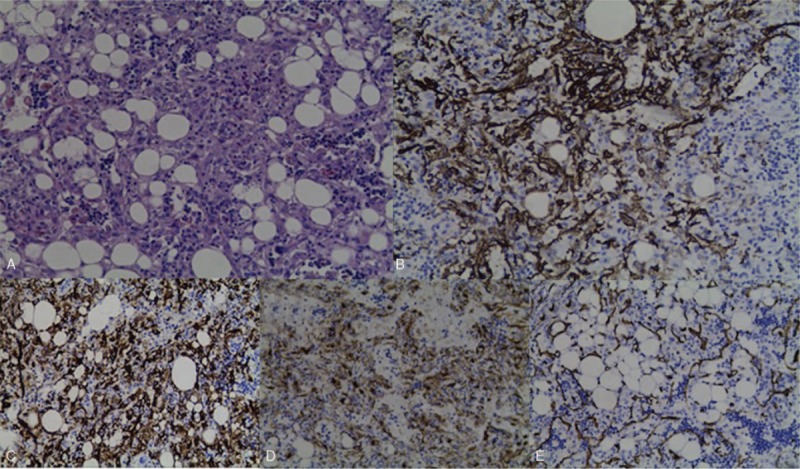
A, Angioleiomyoma tissue stained with H&E (magnification, ×40). B, Angioleiomyoma tissue immunohistochemically stained SMA (B), Melan-A (C), HMB45 (D), CD34 (E) (magnification, ×40).

At this time, enlargement of the hepatic mass was revealed in follow-up. The B-type ultrasonic inspection showed that a hyperechoic nodule could be seen in hepatic left lateral, about 4.5 × 3.9 cm^2^ in size, with a clear boundary, which was considered hepatic hemangioma (Fig. 8). And CT examination showed that in the left hepatic lobe, a kind of nearly-circular low density with a little fat solid density shadow can be seen, with a maximum diameter of 3.8 cm. After enhancement, the mass was significantly unevenly enhanced in the arterial phase and reduced in the venous phase and the delayed phase. It was considered to be primary hepatic carcinoma (Fig. 9). The MRI reported that a nearly-circular abnormal signal shadow, about 4.4×4.2 cm^2^ in size, was seen in hepatic left lateral, presenting a lamellar high signal in phase, a low signal out phase, and an equal or low signal in T2 fat-suppressed imaging. It was considered as liver cancer with steatosis (Fig. 10). Considering the possibility of liver cancer, laparoscopic hepatic left lateral lobectomy was performed. The hepatic mass after excision was about 5 cm. Pathological examination reported that the tumor tissue consisted of many adipose tissues, epithelioid cells, and thick-walled blood vessels, and a large amount of lymphocyte infiltration was seen in the tumor stroma. And the results of the immunohistochemical analysis showed as follows: HMB45 (positive), Melan-A (positive), S-100 (focal Positive), Ki-67 (5% Positive), CD34 (vascular Positive), SMA (Positive), Desmin (Negative), Hepatocyte (Negative), CD68 (focal Positive), P53 (Negative), Vim (Positive), CD117 (Negative) (Fig. 11). According to the pathological features described above, the mass of the liver was diagnosed as hepatic angioleiomyoma. From the first kidney surgery to the rehospitalization, the follow-up time was 2 years, and the follow-up time was 4 months after the liver surgery during the second hospitalization. The patient's tumor did not relapse and no other surgical complications occurred.

## 5 Discussion

Preoperative diagnosis of angioleiomyoma is very difficult and usually depends on postoperative or biopsy pathology. Angioleiomyoma tissue was visible under the microscope a larger number of the smooth muscle fiber bundle, mixed with rich thick wall vessel structure, but without common malignant tumor cell nuclear atypia or mitotic significantly, and without hemorrhage or necrosis in the tumor. Observed by a microscope, angioleiomyoma was a well-circumscribed and encapsulated tumor characterized by a larger number of intervascular bundles of smooth muscle fiber surrounding multiple thick vascular spaces of varying size. But no nuclear atypia or mitotic figures were identified.^[3–5,9–13]^ According to the ratio of smooth muscle cells to vascular cavity structure, angioleiomyoma can be classified into 3 subtypes: capillary or solid, cavernous, and venous.^[2–5,9–13]^ Solid angioleiomyoma is characterized by small diameter or fissure-like vascular in the tumor that is surrounded by many differentiated mature smooth muscle cells. In some cases, calcifications or adipose tissue can be seen. The venous tumors are mainly composed of thick-walled venous vessels surrounded by the ring or swirl smooth muscle cells, and the smooth muscle cells outside the vascular wall and in the mesenchymal are transitional. For cavernous tumors, there are more expansion of cavernous blood vessels and different thicknesses of smooth muscle fibers around the blood vessel wall. And there are mature connective tissue collagen fibers around the tumor stroma, which may be a myxoid or hyaline degeneration but no obvious fibroblasts and inflammatory cells.^[13]^ Immunohistochemically staining is helpful for the pathological diagnosis of vascular leiomyoma. SMA is strongly positive in intravascular and smooth muscle fibers intervascular. CD34 is strongly expressed in vascular endothelial cells, but Vimentin expression is different in different patients.^[12,14]^

Angioleiomyoma occurs predominantly in people between the ages of 40 and 60. The lesions that occur in the soft tissue of the limbs and head and neck are mostly small (diameter is less than 2 cm predominantly) and are mostly manifest as skin or subcutaneous nodules and may have pain and/or tenderness.^[1,5,7]^ The clinical manifestations of vascular leiomyoma occurring in the internal organs are related to the location and size of the tumor. It can be manifested as abdominal pain, mass, bleeding, etc. Neither of the 2 patients in this study had obvious clinical symptoms and was hospitalized for liver masses, which was proved to be hepatic angioleiomyoma by postoperative pathology.

Since Beissert et al^[1]^ reported the first adult hepatic angioleiomyoma in 2002, there have been few reports on hepatic angioleiomyoma for retrieval. Our recent study had the following unique features: One of the patients had both kidney and liver angioleiomyoma, which was rare in the internal organs and even rarer in 2 different organs at the same time. The patient in case 2 had been diagnosed with liver tumors and left kidney angioleiomyoma 2 years earlier. However, the pathological diagnosis of the nature of liver masses was not made at that time. Since there was no pathological diagnosis at the time, we chose to follow up the liver mass regularly. This treatment strategy may harm the patient's benefit if the liver tumor is malignant. This case will serve as a warning for future clinical decisions. From the first discovery of diseases in 2 organs to the resection of liver neoplasm in this operation, the angioleiomyoma in the kidney of the patient did not relapse during the follow-up period of about 2 years, and the angioleiomyoma in the liver did not metastasize, and there were no new tumors in other organs or tissues, which also verified that the angioleiomyoma was benign tumor.

For hepatic angioleiomyoma, some of them are similar with hepatic hemangioma in terms of imaging manifestations, while others are similar with liver cancer but not completely consistent with liver cancer and hepatic hemangioma. The proportion of smooth muscle cells, fibrous tissue, vascular cavity, and necrotic tissue in the lesion may result in a nonspecific imaging appearance of vascular leiomyoma in a way. Two patients in this study, one was considered for hepatic hemangioma preoperative, the other for hepatocellular carcinoma or hepatic hemangioma. In practice, angioleiomyoma is benign lesions in the liver that can be cured by complete resection according to the surgery principle of hepatocellular carcinoma or hepatic hemangioma, even if not confirmed by preoperative imaging examination,

All in all, the liver angioleiomyoma is a rare tumor, with extremely low incidence, so it is difficult to collect the analysis summary of most cases. And because of atypical clinical symptoms and no specificity in laboratory examination, lack of characteristic imaging findings, angioleiomyoma is easily misdiagnosed for another liver disease. Clinically, it should be differentiated from other liver tumors, such as hepatic hemangioma, hepatic angiolipoma, hepatic lymphoma, etc. Because of the rarity of the hepatic angioleiomyoma, there are no retrospective studies or randomized controlled studies comparing the merits of different treatments. Combined with the treatment of the above 2 cases and other clinical reports, the author believes that regular follow-up is acceptable if the preoperative pathological diagnosis can be made clearly and the patient has no other symptoms. And if the pathological diagnosis cannot be determined preoperatively, that is, the possibility of a malignant tumor cannot be completely excluded or the patient has clear symptoms of discomfort, we should choose surgical treatment. But in terms of treatment strategy, we are more inclined to choose surgery because surgical treatment is usually better.

## Acknowledgments

All the patients included in this article and all medical personnel involved in disease diagnosis and treatment should be thanked.

## Author contributions

**Data collection:** Jian-Bo Xu, Ya-Bin Yu

**Resources:** Jian-Bo Xu, Ya-Bin Yu.

**Writing – original draft:** Bin Jiang, Qiuni Chen.

**Writing – review & editing:** Fu-Zhen Qi, Yan Song.
